# Investigating eco-evolutionary processes of microbial community assembly in the wild using a model leaf litter system

**DOI:** 10.1093/ismejo/wrae043

**Published:** 2024-03-20

**Authors:** Kristin M Barbour, Jennifer B H Martiny

**Affiliations:** Department of Ecology and Evolutionary Biology, University of California, Irvine, Irvine, CA 92697, United States; Department of Ecology and Evolutionary Biology, University of California, Irvine, Irvine, CA 92697, United States

**Keywords:** community assembly, eco-evolutionary processes, microbial communities, soil, microbiomes, decomposition, Curtobacterium

## Abstract

Microbial communities are not the easiest to manipulate experimentally in natural ecosystems. However, leaf litter—topmost layer of surface soil—is uniquely suitable to investigate the complexities of community assembly. Here, we reflect on over a decade of collaborative work to address this topic using leaf litter as a model system in Southern California ecosystems. By leveraging a number of methodological advantages of the system, we have worked to demonstrate how four processes—selection, dispersal, drift, and diversification—contribute to bacterial and fungal community assembly and ultimately impact community functioning. Although many dimensions remain to be investigated, our initial results demonstrate that both ecological and evolutionary processes occur simultaneously to influence microbial community assembly. We propose that the development of additional and experimentally tractable microbial systems will be enormously valuable to test the role of eco-evolutionary processes in natural settings and their implications in the face of rapid global change.

## Introduction

Community assembly describes the processes that shape the identity and abundance of organisms in ecological communities [1]. These processes are key to understanding the foundational principles of ecology, including biogeographic patterns, community responses to environmental change, and the relationship between biodiversity and ecosystem functioning [2-4]. Understanding the assembly of microbial communities (or microbiomes) specifically can also facilitate our ability to modify or engineer them to improve human and environmental health [5, 6].

Four processes influence the assembly of ecological communities, microbial, or otherwise: selection, dispersal, ecological drift, and diversification [7-9]. Ideally, one would like to manipulate the influence of each process separately and in combination while allowing community assembly to proceed over many generations, but such experiments are often impractical for plant and animal systems. Some clever experiments have been conducted that, for instance, modify dispersal or drift [10-13], but investigating diversification (i.e. evolution) during community assembly is particularly difficult [8, 14, 15]. Thus, most support for how these four interacting processes come together to shape the composition of ecological communities is derived from observed patterns, theoretical models, or laboratory studies [16-19].

Microbial communities have been useful for testing community assembly theory in the lab (e.g. [20-22]), but they are not the most obvious system for testing these theories in natural ecosystems. Unlike plants, microbes are not easily “seeded” into plots, and unlike animals, they cannot be marked and recaptured. Microbial communities are also orders of magnitude more diverse than their plant and animal counterparts, and many taxa have yet to be cultured and described. At the same time, microbial communities can be easy to replicate and manipulate. Their relatively fast generation times allow for experiments to take place over many generations. And for studying community assembly in particular, an underappreciated advantage of microbial communities is that the ecological and evolutionary processes shaping them often occur simultaneously [23]. With new methods for genome-resolved sequencing, it is therefore possible to track both the ecological dynamics of a diverse microbiome and, simultaneously, the evolution of many “species” within it.

**Figure 1:**
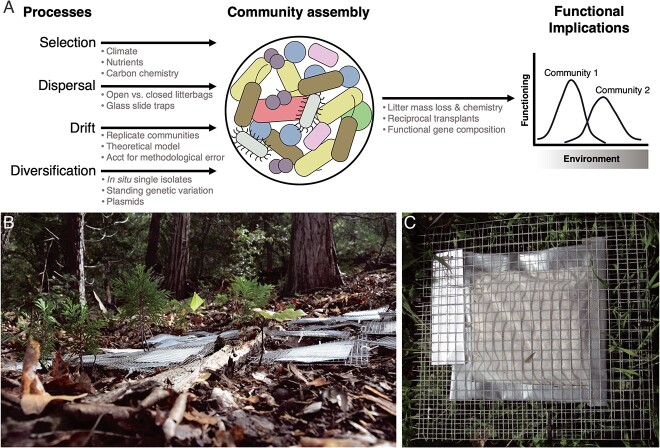
Leaf litter as an experimental system in the field; (A) an overview of the four processes, their links to microbial community assembly and functioning, and the types of experiments and measurements that we have used to investigate each link; (B) leaf litter-containing bags, or microbial “cages,” on the soil surface of a pine-oak and (C) grassland ecosystem; the cages are made from nylon mesh that prevents microbial dispersal and placed in larger metal screening to prevent animal disturbance.

Here, we review more than a decade of collaborative efforts to study microbial community assembly in the field. We first summarize some of the benefits of our model system, the leaf litter layer of surface soil in Southern California ecosystems. Our field methods are easily deployable (involving nylon mesh, duct tape, hair straighteners, and coffee grinders!) and repeatable, as confirmed by trial and error over many years. Then, we present evidence for each of the four community assembly processes working alone to influence microbial composition and, sometimes, in concert (Fig. 1A). Finally, we discuss the implications of these results for ecosystem functioning and suggest future directions for research. We hope that these initial results provide inspiration for possibilities in other systems with their own unique advantages while recognizing that parallel efforts by other researchers are already ongoing. After all, we will need a variety of experimental systems where eco-evolutionary processes can be iteratively examined and manipulated to develop a general understanding of microbial community assembly and its implications in the face of global environmental change.

## Leaf litter microbiome as a model for community assembly

Decomposition of leaf litter is an essential component of terrestrial carbon and nutrient cycling, largely governed by microorganisms. The leaf litter layer is the collection of dead and decaying plant biomass (leaves, shoots, and woody debris) that makes up the topmost layer of soil. Leaf litter influences the bulk (mineral) soil below, altering abiotic properties, such as light, moisture, and temperature. During decomposition, microorganisms both mineralize carbon compounds in leaf litter, producing CO_2_, and contribute to the production of stable, recalcitrant soil organic matter through plant biomass processing and necromass formation [24, 25]. Microbial degradation also releases nutrients into the surrounding bulk soil, altering resource availability for plants and soil fauna and mediating the flow of carbon and nutrients from the surface into deeper soil [26]. In the temperate ecosystems where we work, the litter layer is seasonally dynamic. A large pulse of litter accumulates at the end of the wet season and then slowly decays throughout the rest of the year. The physical architecture of the leaf litter layer varies greatly across ecosystem types. In forests and shrublands, fallen leaves constitute a large portion of the leaf litter, whereas in grasslands and some croplands, standing dieback contributes a large amount of litter mass.

Beyond its role in ecosystem functioning, leaf litter has several useful features that lend themselves to studying community assembly. First, it is naturally patchy, allowing the easy application of a metacommunity framework to the system [1]. A community can be defined by a single leaf or patches of leaves connected by dispersal. Second, the leaf litter layer experiences more environmental variation than the bulk soil below. At our field site and in many locations, the soil surface can be quite hostile in terms of UV and moisture stress, and temperature and moisture fluctuate daily and seasonally. Thus, leaf litter communities are likely more sensitive to environmental change and experimental treatments than are communities deeper in the soil profile [27]. Third, the leaf litter layer is readily accessible and naturally replenishes. Repeatedly sampling leaf litter is less destructive than taking soil cores, allowing for longitudinal sampling in the same locations without disrupting the bulk soil structure. Finally, microbial diversity in leaf litter is high but manageable. Both the bacterial and fungal communities are more diverse than laboratory consortia, while less diverse (both in richness and evenness) than those in bulk soil or sediments. Consequently, there is hope of attaining a detailed understanding of the biology of the most abundant members of the community while studying complex community dynamics in the environment.

We have further developed and refined several methods that make leaf litter a practical field system. First, it is relatively easy to measure key ecological metrics of leaf litter compared with bulk soil. For instance, quantifying microbial abundance by microscopy (for fungi) or flow cytometry (for bacteria) is easier than in bulk soil [28]. It is also straightforward to assess decomposition by measuring mass loss from the litter bags over time [29] and to measure potential extracellular enzyme activity and leaf litter chemistry [30, 31]. We have also isolated many of the most abundant bacterial and fungal taxa from our local leaf litter by culturing them on media made from litter leachate. Hence, we can create ecologically relevant consortia [32, 33] and investigate the phenotypic diversity of these taxa [34, 35].

Most importantly for studying community assembly, however, the microbial community in leaf litter can be manipulated separately from the abiotic environment and the litter substrate (which may differ in carbon and nutrient resources, pH, and moisture retention). To do this, we reduce the abundance of the resident community using gamma irradiation and/or autoclaving and then reinoculate the litter with a small amount (1% w/w litter) of an intact field community. Although completely sterilizing the litter is unlikely (and nearly impossible to demonstrate), the procedure successfully “grafts” the inoculum community onto the original litter substrate such that the new community closely matches the inoculum community and not the original community [36]. The community is then enclosed in a mesh litterbag that allows moisture and nutrients to flow through and, depending on the membrane pore size, either blocks or allows dispersal of bacteria, fungi, soil fauna, and larger animals (Fig. 1B and C). These microbial “cages” provide a way to replicate a homogenized inoculum community into replicate patches and transplant them into different environments (treatments or sites) [37].

There are also caveats to using these cages for manipulating the leaf litter community. Although the microbial composition within the bags is similar to the surrounding leaf litter, nylon mesh blocks sunlight and may trap moisture, altering the abiotic environment compared to the surrounding area. In addition, viruses and very small cells may still get into the “closed” cages that aim to exclude microbial dispersal. Similarly, there may be undetectable damage to the integrity of the cages in the field that allows for mixing with nearby communities. For these reasons, we always include controls, such as litterbags that are open to dispersal and/or bags inoculated with the local community, to account for these potential issues.

## Evidence of the four assembly processes at work

Leveraging the methodological advantages of leaf litter, we have conducted a variety of field (and lab) experiments on their microbial communities. Below, we summarize these studies and synthesize key outcomes from this system. Although the assembly processes are highly intertwined, we discuss them separately for organizational purposes.

### Selection

Evolutionary biologists define selection as shifts in allele frequencies within a population due to the differential fitness of individuals. However, this definition of selection can be expanded to the community level, where shifts in the frequencies of species (or taxa or other units) reflect fitness differences among phenotypes [8]. Thus, the effect of selection by abiotic or biotic factors on the relative abundance of microbial taxa, also known as species sorting [38], can be assessed within an entire community.

**Figure 2:**
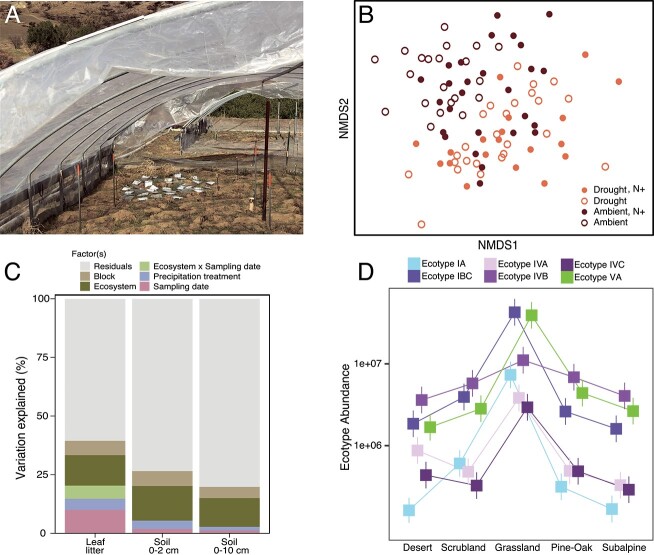
Selection by the abiotic and biotic environment affects leaf litter community assembly; (A) a microbial cage experiment in the Loma Ridge Global Change Experiment (LRGCE) simulated drought plots following a wildfire; polyethylene sheets are pulled over the plots during half of the annual rainfall events at the site to exclude ~50% of the annual precipitation; (B) non-metric multidimensional scaling ordination (NMDS) depicting how bacterial community composition on grassland leaf litter varies across four LRGCE treatment combinations; redrawn from elsewhere [42]; (C) estimated percent variation explained by factors significantly impacting bacterial community composition in the leaf litter layer, top 2 cm of bulk soil, and top 10 cm of bulk soil at the LRGCE; reprinted with permission from [27]; (D) absolute abundances (by cell count) of the most abundant *Curtobacterium* ecotypes (±1 SD) in the leaf litter layer at five sites across a climate gradient; redrawn from elsewhere [58].

In leaf litter, we have focused on how selection by environmental change may influence microbial community assembly. Our main site for studying this has been the Loma Ridge Global Change Experiment (LRGCE) in Irvine, CA, USA. This experiment was established in 2007 to simulate the increased frequency of drought and nitrogen availability in two dominant ecosystem types (a semi-arid grassland and coastal sage scrubland, CSS) in the area [39]. We have characterized the effects of the experimental treatments on the bacterial and fungal communities in leaf litter from these plots for over a decade (Fig. 2A).

Simulated global changes select for distinct bacterial and fungal communities in these ecosystems, as observed previously in many other experiments [40, 41]. Drought, nitrogen addition, and their interaction alter microbial community composition in the leaf litter (Fig. 2B) as well as the bulk soil [27, 42, 43], even when controlling for differences in litter substrate and its successional stage. Further, the responses of the microbial community to global change depend on the plant community [43]. Drought does not select for the same microbial taxa within the grassland as it does within the CSS, although these ecosystems are immediately adjacent to one another and experience the same climate. This interactive effect thus indicates that the response to drought is mediated by biotic resources, including, for instance, the chemical composition of the leaf litter [44, 45]. As a result, some global change responses may be difficult to transfer between ecosystems [46]. In contrast, bacterial community assembly after a wildfire at the LRGCE did not depend on the ecosystem or precipitation history [47]. Instead, wildfire was selected for known, fire-loving taxa, including the bacterial genus *Massilia* [48, 49].

We have further sought to understand the importance of global change relative to other factors that influence microbial community assembly. In fact, a large amount of community variation within our site can be attributed to seasonal or interannual variation (~10%–39%), i.e. likely driven by a combination of fluctuations in temperature, moisture, UV, and the successional stage of the leaf litter (Fig. 2C) [27, 42]. In comparison, simulated global changes have a more modest effect on microbial community composition in the leaf litter. For instance, across our studies, drought consistently explains ~4% of variation in bacterial composition, although the strength of this effect increases to 10% if interactive effects (i.e. drought × time and drought × ecosystem) are also considered [27, 42, 43]. Temporal variability in aquatic systems has long been recognized [50, 51], yet such high intra- and inter-annual variability in surface soils has been less studied. This bias may be partly due to the disruptive nature of taking soil cores from the same plot over time, leading soil researchers to be cautious about the number of samples they collect. Thus, another benefit of the leaf litter system is that its regenerative nature lends itself to long-term longitudinal sampling.

This large “background” of temporal variability in our system has several important implications for investigating community assembly processes. First, it means that selection by a treatment can be missed without enough replicates and/or longitudinal sampling due to a lack of statistical power. Second, it suggests that the effects of global change factors may vary over time. For instance, the timing of a drought—whether it occurs during a wet or dry year or season—may alter its effect on community assembly. Indeed, we often detect an interactive effect between drought and sampling time on the composition of the leaf litter community [42, 43]. Third, this background variation is itself context-dependent. The impact of environmental change on the soil communities at our site—whether drought, wildfire, or more generally, temporal variability—is strongest in the leaf litter layer and weakens with soil depth (Fig. 2C) [27]. Altogether, these results highlight the context-dependent of selective forces on community assembly.

Moving forward, a goal is to understand the effects of selection on microbial community composition in a more mechanistic way: can we predict the results of community assembly under different abiotic and biotic conditions? Although we typically measure composition in terms of taxa or other units of biodiversity, selection ultimately increases or decreases the abundance of a taxon because of its traits, or characteristics. Thus, one approach that may provide a more predictive understanding of community responses to particular conditions is to identify the key traits under selection [52-54]. Unfortunately, it is not a simple task to identify which traits matter under a particular selection regime [54]. Toward this end, we have focused on an abundant leaf litter bacterium, *Curtobacterium* (family *Microbacteriaceae*, phylum *Actinomycetota*), that is globally distributed [55] and easily cultured. Sequencing of our isolates revealed extensive genomic diversity that clusters into clades and subclades within those clades [56]. Yet traditional classification methods fail to capture this diversity; all *Curtobacterium* genomes would collapse into two OTUs (defined at 97% 16S rRNA gene sequence similarity) or four exact sequence variants (ESVs) [57]. Physiological assays also revealed that clades within the genus could be distinguished by their ability to degrade carbon sources, form biofilms, and grow under different temperatures [58]. We therefore hypothesized that these traits would relate to the ability of the strains to survive and reproduce on leaf litter across a range of temperatures and moisture stresses.

The combination of genomic and physiological data allowed us to designate *Curtobacterium* ecotypes [58], defined as highly similar genotypic and phenotypic strains that occupy the same ecological niche [59, 60], a concept somewhat comparable to that of a eukaryotic species. The ecotypes further vary in their biogeographic distribution at sites along a climate gradient (Fig. 2D) [58]. After controlling for the litter substrate using our microbial cages, *Curtobacterium* traits correlate with the climate gradient, indicating that both climate conditions and litter substrate select for the composition of ecotypes present at a site [57]. Thus, even though predicting the selective effects on microbial community composition is still overwhelming, it is not inexplicable. A focus on a subset of diversity, combined with experimental manipulation, allowed us to identify traits that underlie climatic responses.

### Dispersal

Compared to selection, the role of dispersal in microbial community assembly remains less clear [22, 61]. Biogeographic patterns provide indirect evidence that dispersal—defined broadly as the movement of organisms across space—might shape microbial composition [9, 62, 63], but more direct evidence is desirable. Given their ease of manipulation, microorganisms have been extensively used in lab experiments to test the influence of dispersal on community assembly, microbial or otherwise. Indeed, many studies demonstrate that dispersal has the potential to be a powerful force in community assembly (e.g. [64, 65]). However, the details of these experiments, including the rate and composition of the individuals dispersing, are often somewhat arbitrary [66].

**Figure 3:**
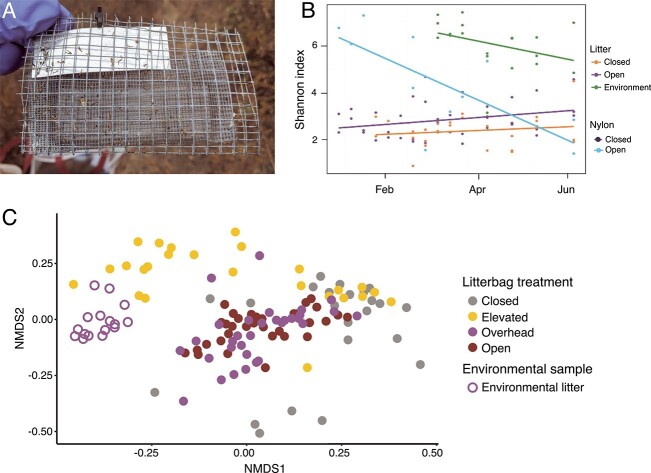
Dispersal from different routes alters microbial communities in the field; (A) glass slide “trap” used to capture microorganisms dispersing into the soil surface; (B) effects of dispersal limitation on bacterial evenness from a field experiment, and line color represents the treatment type: litterbags closed to dispersal (orange), litterbags open to dispersal (purple), nylon-containing bags open to dispersal (light blue), and leaf litter collected from the surrounding environment (green); reprinted from elsewhere [69]; (C) NMDS of bacterial composition in litterbags exposed to different dispersal routes in a grassland (closed = no dispersal; elevated = dispersal from air; overhead = dispersal from air and surrounding environmental litter; open = dispersal from air, environmental litter, and bulk soil); reprinted from elsewhere [70].

Experiments are thus needed to assess the impact of microbial dispersal in natural systems. Leaf litter is a particularly interesting system to study dispersal given the regular inputs of new resources (freshly fallen plant biomass) and its location at the soil-atmosphere interface. A first step to measuring the impact of dispersal on a community is to test the effect of removing it (e.g. [67, 68]). In leaf litter, we can accomplish this by altering the mesh size of the litter bag to compare microbial community assembly in closed litter bags (0.22-μm pores) versus open litter bags (window screen or 18-μm pores to exclude some fungi) (Fig. 3A). Using this approach, we find that dispersal consistently alters the richness, evenness, and composition of the leaf litter microbial community (Fig. 3B) [37, 69]. Furthermore, dispersal and selection can interact to alter leaf litter composition [37]. For instance, dispersal significantly contributed to the reassembly of bacterial and fungal communities after a wildfire, but this effect depended on the ecosystem [47].

Although these results provide *in situ* evidence that dispersal contributes to microbial assembly, they do not consider which microbes are dispersing, from where, and how fast. Details of these rates and routes are needed to develop a deeper understanding of microbial dispersal and how it interacts with other assembly processes. Given that tracking the movement of individual microbes in the field is impractical, we deployed sterile glass slides as microbial “traps” (Fig. 3B). In this way, we can quantify the rate and composition of microorganisms landing on the slides [66]. At the LRGCE, we observed an average of 7900 bacterial cells/cm^2^ immigrating daily into the soil surface and found distinct communities dispersing via different routes, defined as a combination of the source community (e.g. air or soil) and the physical vector (e.g. rain or wind) [70]. Furthermore, exposure to different dispersal routes altered the succession of the microbial community (Fig. 3C) [70].

Altogether, this collection of experiments reveals that dispersal not only contributes to community assembly, but —like selection—is context dependent. For instance, dispersal into the surface leaf litter from the bulk soil appears to be minimal at our site but became more important after wildfire removed the surface litter layer [47]. Moreover, the effects of dispersal on composition are highest during the early stages of litter succession such as after a wildfire or as a green leaf senesces.

### Drift

Ecological drift is the process by which random changes in species relative abundances lead to diversity. Although thought of as its own process, drift is intricately connected to selection and dispersal. Specifically, the impact of drift on community assembly is thought to increase with weak selection pressures and low dispersal [8, 71]. Thus, disentangling the impact of drift from other assembly processes is challenging in ecological communities [72].

**Figure 4:**
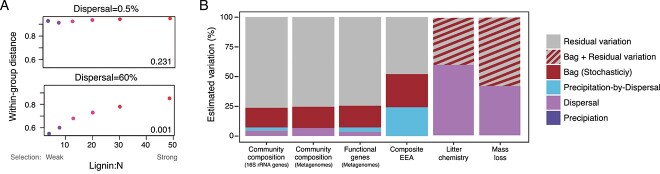
*In silico* and experimental evidence that drift contributes to microbial community assembly; (A) dissimilarity (within-group distance) of replicate communities within lignin/*N* treatments that were exposed to different dispersal rates for 6 years simulated by the Decomposition Model of Enzymatic Traits (DEMENT); the bottom right of each panel shows *P*-values for the null hypothesis that within-group distances across lignin/*N* treatments are equal in a single dispersal level; stochasticity increases (higher within-group distance) with low dispersal rates and stronger selection (higher lignin/*N* values); adapted from elsewhere [73]; (B) estimated percent variation of bacterial community composition (assayed by 16S rRNA gene amplicon and metagenomic sequencing) from litterbags in the field explained by a precipitation treatment, dispersal, and their interaction; within-bag variation (stochasticity) and unexplained (residual) variation were also estimated for ecosystem functioning metrics including extracellular enzyme assays (EEA), litter chemistry, and litter mass loss; reprinted from elsewhere [37].

Given these challenges, we first investigated the role of ecological drift in leaf litter communities using a theoretical model [73]. The Decomposition Model of Enzymatic Traits (DEMENT) simulates microbial communities that produce extracellular enzymes and decompose decaying litter [74]. The impact of drift was assessed by quantifying the degree to which random differences in births, deaths, and dispersal affected the composition of simulated communities. Lower dispersal rates led to higher levels of stochasticity (i.e. higher compositional variation among modeled communities). However, drift also played a large role under high dispersal rates when selection pressure was also high. Communities on chemically complex litter substrates were more susceptible to drift because this highly selective environment reduced total microbial abundance (Fig. 4A) [73].

To move from theory to the field, we next aimed to quantify the effect of ecological drift on leaf litter communities by eliminating the effect of other processes, specifically selection and dispersal. Just as evolutionary biologists measure the effect of genetic drift on populations using highly controlled laboratory experiments [75], we can use our litter bags to minimize confounding field variables to “isolate” the effects of stochastic variation—something that is challenging to do in most other systems [37]. To reduce biological heterogeneity, we inoculated a homogenized microbial community into multiple litter bags filled with irradiated leaf litter. Then, to reduce environmental heterogeneity, we deployed the litter bags within a small (1 m^2^) area at the LRGCE site. Parallel to the theoretical experiments described above, we further manipulated dispersal (open and closed litterbags) and the selective environment (added water versus ambient rainfall) to test whether drift interacts with dispersal and selection, as observed in the theoretical model [73].

Using this highly controlled field litterbag experiment, we found that stochasticity (ecological drift, potentially amplified by priority effects) influenced bacterial community assembly, contributing three times more to compositional variation than dispersal [37] (Fig. 4B). Contrary to our model, however, stochasticity (as quantified by beta-diversity) decreased rather than increased with reduced dispersal. Further, the effects of drift were not restricted to taxonomic composition but also permeated to impact other key aspects of the community, including functional potential and extracellular enzyme activity. We also found that much of the measured variation among replicates could be attributed to methodological factors, such as technical error and spatial heterogeneity within bags; this residual variation accounted for ~75% of the observed variation in community composition (Fig. 4B). This result highlights that the effect of drift on microbial community assembly will be overestimated if these sources of variability are not quantified.

### Diversification

The fourth process of community assembly, diversification, is often mentioned but rarely investigated within the time frame of an ecological study. Even though bacteria can evolve quite rapidly, entirely new bacterial “species” (as measured by the divergence of the 16S rRNA gene) will not emerge for millions of years [76]. Nonetheless, evolution may be occurring within a microbial community at a finer genetic resolution. However, detecting these changes among hundreds or thousands of microbial species within a microbiome is a challenge. Thus, the ability for microbes to evolve, let alone adapt, on ecological timescales remains largely unexplored in natural ecosystems.

**Figure 5:**
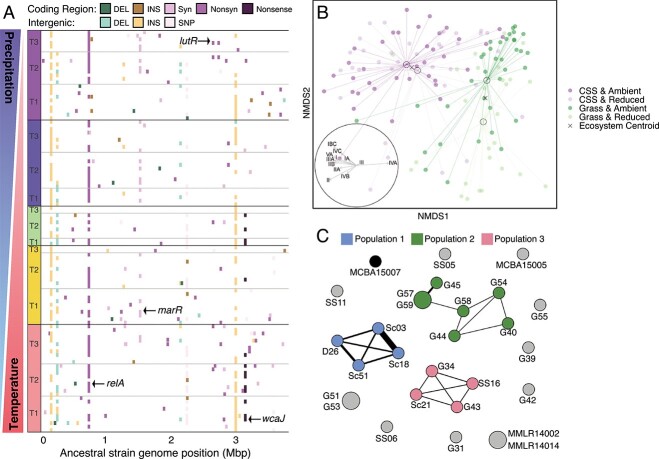
Rapid evolution of a leaf litter bacterium in the field; (A) mutations identified in a *Curtobacterium* strain that was transplanted in litterbags across a climate gradient (red = desert, orange = scrubland, green = grassland, blue = pine-oak, and purple = subalpine), and mutations in 112 evolved strains isolated from five sites along the climate gradient at 6 (time point 1), 12 (T2), and 18-month (T3) intervals, and nonrandom mutations also observed in the population (metagenomic) data are denoted for synonymous (syn), nonsynonymous (nonsyn), and nonsense mutations; reprinted from elsewhere [57]; (B) NMDS of *Curtobacterium* microdiversity (ESVs of the *groEL* gene) from grass litter collected from ambient and reduced precipitation plots in the grassland and CSS at the LRGCE; centroids of each ecosystem × precipitation treatment combination are marked by black circles, and the centroids of all samples from each ecosystem are marked by an X; inset indicates the direction and strength of correlation with *Curtobacterium* subclades; reprinted from elsewhere [80]; (C) recombination network across all pairwise combinations of 26 *Curtobacterium* strains; thicker edges represent increased recombination between strains; nodes are colored by population designation where populations are defined as groups with the potential to exchange genetic material; node size indicates the number of clonal clusters (strains too closely related to differentiate recombination); reprinted from elsewhere [82].

To investigate the potential for rapid evolution in leaf litter microbial communities, we first asked whether we could detect the emergence of *de novo* mutations. Once again, we used our microbial cages to conduct a field experiment. Mirroring laboratory evolution experiments [75, 77], we inoculated replicate litter cages with a single isogenic *Curtobacterium* strain. We then deployed the cages across an elevational gradient of temperature and precipitation. Every 6 months, we reisolated bacterial colonies from each cage and identified a variety of nonrandom, parallel single nucleotide polymorphisms (SNPs) that we confirmed with metagenomic sequencing. SNPs were found in genes related to nutrient acquisition, stress response, and exopolysaccharide production (Fig. 5A) [57]. These mutations provide a new source of genetic diversity that might allow for adaptation, but further work is needed to determine if these mutations impact organismal fitness.

Evolution occurs not only through new mutations but also through shifts in standing genetic variation within a population. Microbial species, or ecotypes, encompass standing genetic variation that often coexists within an ecosystem [78, 79]. This so-called “microdiversity” is also observed within *Curtobacterium* in leaf litter. To track this finer diversity, we developed genus-specific primers for a protein-encoding gene (groEL). *Curtobacterium* microdiversity—here, the relative abundance of ESVs of the *groEL* gene—responded to selection by drought and the litter substrate within the global change experiment (Fig. 5B) [80]. Thus, responses at this fine level of genetic resolution reflect shifts in allele frequencies, a phenomenon that, among larger organisms, would be thought of as an evolutionary process [81].

The evolutionary process that we arguably know least about within microbial communities is recombination. This is an unfortunate gap, as gene flow, or the exchange of genetic variation, is what delineates populations, which are often considered the fundamental unit of evolution. Using a collection of *Curtobacterium* isolates from across Southern California, we identified at least three recombining populations of *Curtobacterium* within one subclade of an ecotype [82]. The populations were delineated using gene flow discontinuities, where we quantified signals of increased “recent” recombination among strains that were clustered into discrete populations (Fig. 5C) [83]. Strains within a population shared more flexible genes than expected by chance, and recombination of population-specific genes appeared to be mediated by homologous recombination. Bacteria can also exchange genes via horizontal gene transfer by plasmids, a pattern we also observed among our *Curtobacterium* isolates. Using long-read sequencing of our isolates, we identified numerous plasmids that vary greatly in their size and genetic content, even among very closely related isolates [84]. The plasmids encode a diversity of traits that are not a random subset of chromosomal traits, ranging from genes involved in carbon and nitrogen cycling to cell motility. Yet, the time-scale upon which recombination (through homologous replacement of short gene segments or transfer of an entire plasmid) contributes to *Curtobacterium* diversity in leaf litter remains unclear. We do not yet know how often strains are exchanging genetic information—either via plasmids or recombination—in leaf litter for the observed patterns to emerge.

Overall, zooming into just a single bacterial genus allowed us to highlight the potential for rapid evolution to influence genetic diversity in the leaf litter microbiome. This picture is admittedly still limited. We have yet to assess the time-scale of recombination, including horizontal gene transfer, within soil microbial communities, despite its inferred importance for microbial adaptation [85]. These details are needed to provide a holistic understanding of the potential for microbial communities to adapt to future environmental change. The answer, for instance, could depend on the relative importance of diversity generated by rapid evolution versus that contributed through dispersal [19, 76].

## Implications for community functioning

Our work on leaf litter illuminates how a range of ecological and evolutionary processes can contribute to the assembly of environmental microbiomes. These results in themselves provide useful examples for the generation and maintenance of diversity within microbiomes and ecological communities more generally. Yet the question remains: does community assembly lead to communities that are functionally distinct?

**Figure 6:**
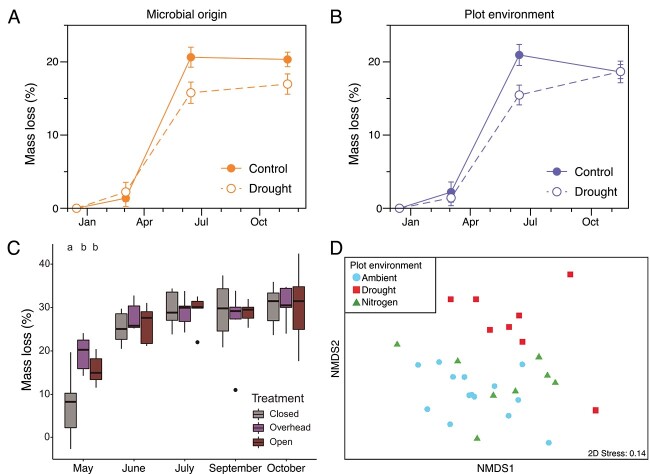
Differential assembly of leaf litter microbial communities impacts decomposition; effect of (A) microbial origin and (B) contemporary plot environment on percentage mass loss in litterbags during the first year of a reciprocal transplant experiment, where microbial origin refers to leaf litter community inoculum that was exposed to either ambient (control) or reduced (drought) precipitation at the LRGCE; adapted from elsewhere [29]; (C) mass loss of leaf litter closed to dispersal in the field compared with litter exposed to all dispersal (open) and litter exposed to dispersal from above the soil surface (overhead); exposure to dispersal accelerated leaf litter decomposition in the first month of the experiment; reprinted from elsewhere [70], and (D) NMDS depicting that the drought, but not added nitrogen, treatment altered glycoside hydrolase composition of the bacterial communities on leaf litter; redrawn from elsewhere [87].

Thus far, we have limited ourselves to discussing the results of our experiments as they pertain to community assembly. However, in many of the studies, we also measured functional metrics of decomposition, allowing us to also address the idea of functional redundancy. Specifically, the microbial cages allow us to disentangle the influence of the abiotic environment from the initial microbial community composition on functional outcomes, which we measure later in an experiment [86]. For instance, we found that drought communities (leaf litter communities assembled under drought conditions at the LRGCE) altered litter decomposition rates separately from the abiotic effect of drought itself (Fig. 6A). Indeed, the effect of community composition was as large as the abiotic effect of drought (Fig. 6B) [29, 87]. Similarly, leaf litter communities assembled along a climate gradient in Southern California decomposed litter at different rates when transplanted to a common environment along the gradient [36]. Furthermore, the resulting chemistry of the decomposed leaf litter depended on the initial microbial inoculum, revealing that different communities utilize unique sets of compounds in the litter. Both of these studies demonstrate that selection altered community assembly and, in turn, resulted in functionally divergent communities.

The processes of dispersal and drift can also influence the functioning of leaf litter communities. Communities exposed to dispersal initially decomposed leaf litter more than twice as fast as communities closed to dispersal, an effect that dampened during later stages of leaf decomposition (Fig. 6C) [70]. And, as previously mentioned, ecological drift not only impacted taxonomic composition but also permeated to impact functional potential and extracellular enzyme activity (Fig. 4B) [37].

We would also like to understand the particular traits in a community that are responsible for changes in overall functioning. In the lab, leaf litter bacteria vary widely in their use of simple carbon substrates and the rate at which they decompose complex leaf litter [34]. In the field, metagenomic sequencing reveals that nitrogen cycling genes and carbohydrate degradation genes vary between the global change treatments at the LRGCE [31, 88]. In one particular experiment, we examined the composition of glycoside hydrolase (GH) genes that degrade different polysaccharides in leaf litter bags transplanted into the different LRGCE treatments. Drought, but not nitrogen addition, shifted GH gene composition, suggesting a mechanistic reason for why decomposition was more resilient to changes in nitrogen than to changes in rainfall (Fig. 6C) [87].

## Moving forward

By focusing on the leaf litter system, we have derived a more detailed understanding of the drivers and context-dependency of the processes underlying microbial community assembly. However, despite many years of work, there are still large gaps in our knowledge about this one system. In particular, we have focused on bacteria and (some) fungi but have neglected the impact of macro- and microfauna that breakdown larger fragments of leaf litter and potentially disperse microorganisms in the field. Some of these organisms, such as nematodes, also graze on microbes and thus may considerably impact the microbial community [89, 90]. For instance, shifts in microbivore composition contributed to the differential assembly of microbial communities exposed to dispersal than those that were not [91, 92]. We have also nearly completely ignored how microbe-microbe interactions, including synergistic and antagonistic interactions between bacteria and fungi, impact community assembly [93, 94]. Similarly, bacteriophages may modify bacterial communities through predator–prey interactions and are known to be abundant and dynamic in ecosystems like ours [95].

Thus far, we have primarily focused on quantifying the effects of one process at a time; however, they will co-occur and likely interact. In particular, although we have only begun to explore the role of contemporary evolution in community assembly in leaf litter, it is clear that ecological and evolutionary processes occur simultaneously. Selective forces such as those imposed by global environmental changes shift allele frequencies within *Curtobacterium* species and the frequency of other, broader taxa in the community. And, just as we have observed interactions between ecological processes [37], we expect that evolutionary and ecological processes will also interact, resulting in eco-evolutionary feedbacks [96]. Microbial communities thus offer an opportunity to test the role of eco-evolutionary feedbacks in natural settings and, with further advancement of genome-resolved tools, assess their effects at different biological scales of organization within the same community. Given the central role of microorganisms in ecosystem functioning, these dynamics may indeed be important for climate feedbacks and mitigation [97, 98].
